# T-BET drives the conversion of human type 3 innate lymphoid cells into functional NK cells

**DOI:** 10.3389/fimmu.2022.975778

**Published:** 2022-10-18

**Authors:** Laura Kiekens, Sigrid Wahlen, Eva Persyn, Zenzi De Vos, Tom Taghon, Bart Vandekerckhove, Georges Leclercq

**Affiliations:** ^1^ Laboratory of Experimental Immunology, Department of Diagnostic Sciences, Ghent University, Ghent, Belgium; ^2^ Cancer Research Institute Ghent (CRIG), Ghent, Belgium

**Keywords:** human innate lymphoid cells, ILC3, NK cells, transdifferentiation, plasticity, T-BET, EOMES

## Abstract

Type 3 innate lymphoid cells (ILC3s) are characterized by RORγt expression and they produce IL-22 upon activation. ILC3s play a role in maintenance of barrier integrity in the intestine. Under inflammatory conditions, the ILC composition of the mucosal tissues is altered due to a high degree of plasticity. It has been extensively demonstrated that both murine and human ILC3s convert into ILC1s to mediate appropriate immune responses. However, plasticity between human ILC3s and NK cells is less well documented. As T-BET and EOMES are key transcription factors in NK cell differentiation, we investigated whether ectopic T-BET or EOMES expression converts human ILC3s into NK cells. ILC3s with ectopic T-BET and EOMES expression downregulate RORγt expression, while T-BET-overexpressing ILC3s additionally upregulate EOMES expression. High E ctopic T-BET expression in ILC3s results in transdifferentiation towards CD94^+^ NK cells, whereas ectopic EOMES overexpression results in dedifferentiation of ILC3s into CD94-CD117^-/low^ cells but is ineffective in NK cell generation. Dedifferentiating ILC3s from both T-BET and EOMES overexpression cultures upregulate NK cell receptors, perforin and granzyme B. Finally, IL-22 secretion is completely blocked in transdifferentiating ILC3s with both T-BET and EOMES ectopic expression, whereas only T-BET overexpression increases IFN-γ secretion and cytotoxicity. Altogether, these findings demonstrate that human ILC3s can convert into functional NK cells, wherein T-BET, and not EOMES, is the main driver.

## 1 Introduction

Innate lymphoid cells (ILCs) are a heterogeneous population of lymphocytes lacking recombination activating gene (RAG)-dependent rearranged antigen receptors and are involved in early immune responses and tissue homeostasis. ILCs can be categorized into five subsets based on their phenotypes, secreted cytokines and transcriptional programs that drive their differentiation (1, 2). ILC1s and natural killer (NK) cells, which are defined by their ability to produce IFN-γ, both depend on the transcription factor T-BET for development and function. However, NK cells are cytotoxic cells that additionally express EOMES, while ILC1s are generally non-cytotoxic cells that do not depend on EOMES. ILC2s produce signature cytokines IL-4, IL-5 and IL-13 and are characterized by the expression of GATA3 and prostaglandin D2 receptor CRTH2. ILC3s are defined by the expression of RORγt and can be subdivided on the basis of the expression of natural cytotoxicity receptors. In humans, NKp44^-^ILC3 produce IL-17 while NKp44^+^ILC3 are strong IL-22 producers. Like ILC3s, Lymphoid Tissue inducer (LTi) cells are uniquely dependent on RORγt for development and secrete IL-17 and IL-22. However, they are a distinct population involved in the lymphoid organogenesis during development (1–4).

In steady state, ILCs are predominantly found in mucosa-associated tissues at barrier surfaces where they rapidly respond to environmental stimuli and various pathogens. ILC1s and NK cells are involved in the control of intracellular bacteria and NK cells further limit viral infections and tumor cells. ILC2s play a physiological role in the defense against helminth infections, airway remodulation and skin repair. ILC3s help to constrain extracellular bacteria and fungi and are mainly localized in the intestinal lamina propria where they play a role in intestinal homeostasis and maintenance of barrier integrity (4, 5). In order to shape an appropriate immune response, mature ILC populations have the ability to acquire the functions and phenotype of other mature ILC subsets and thereby markedly change the ILC composition of a tissue without the recruitment of other cells. This is the concept of ILC plasticity, which is well known in both mice and human (5–7). It was demonstrated that murine IL-22 producing ILC3s can convert into IFN-γ producing ILC1s in response to IL-12 or upon inflammation in the murine intestine (8–10). In humans, the conversion of ILC3 into ILC1 is also driven by IL-12, which induces downregulation of RORγt and upregulation of T-BET expression *in vitro*. Moreover, in the inflamed areas of the intestines of patients with Crohn’s disease, ILC1 numbers are drastically increased, while ILC3 numbers are diminished and total ILC numbers remain unaltered. This suggests that chronic inflammation results in ILC3 to ILC1 transdifferentiation, wherein ILCs possibly contribute to pathology (11, 12). More recently, intermediate populations between ILC3s and ILC1s have been identified in the human tonsil and small intestine by single cell RNAseq analysis, providing evidence for ILC3 to ILC1 transdifferention *in vivo* (13). ILC3-ILC1 plasticity is also a reversible process as IL-1β and IL-23 can reconvert ILC1 into ILC3 that produce IL-22. This suggests that after resolution of inflammation, ILC1s can convert back into ILC3, the main population under steady state (12). Furthermore, there is evidence of ILC2 conversion into ILC1 under the influence of IL-1β and IL-12 *in vitro*. *In vivo* observation of ILC2-ILC1 plasticity was found in the lungs of mice infected with influenza virus (14). Evidence of ILC2 to ILC1 conversion in humans was found in intestines of patients with Crohn’s disease and in the lungs and blood of patients with chronic obstructive pulmonary disease (15, 16). ILC2-to-ILC1 plasticity is also a reversible process and is induced by IL-4 (16). Finally, plasticity between ILC1 and NK cells was observed in the tumor microenvironment, wherein TGF-β plays a crucial role in the development of ILC1s (17, 18). Conversion of NK cells into ILC1 was also seen in mice with non-alcoholic fatty liver disease (19). Yet it is unclear if ILC1 to NK cells transdifferentiation occurs and up to date no evidence exists on ILC1/ILC3 to ILC2 plasticity (7).

As discussed above, transdifferentiation of ILC3s into ILC1s and of NK cells into ILC1s has been well described. In contrast, transdifferentiation of ILC3 into NK cells is far less documented. However, since in humans a RORγt^+^ ILC precursor has been defined that gives rise to both NK cells and ILC3s (20), these populations might be closely related. Here, we investigate ILC3-NK cell plasticity in mature ILC3s with either ectopic T-BET or EOMES overexpression. T-BET and EOMES overexpression in ILC3s results in dedifferentiation into CD94^-^CD117^-/low^ cells, wherein RORγt expression is downregulated. Moreover, high ectopic T-BET expression also results in transdifferentiation towards genuine NK cells that express NK cell receptors, cytotoxic effector proteins perforin and granzyme B, and EOMES. The transdifferentiating cells lose the capability to produce IL-22, while gaining IFN-γ producing capacities and cytotoxicity against tumor cells. Mature ILC3s are thus able to transdifferentiate into cytotoxic NK cells, predominantly driven by upregulation of T-BET, wherein CD94^-^CD117^-/low^ cells are not a mandatory intermediate stage of ILC3-NK cell transdifferentiation.

## 2 Materials and methods

### 2.1 Inducible retroviral overexpression constructs

Inducible human T-BET and EOMES overexpression constructs were obtained by fusing human T-BET or EOMES cDNA (Source BioScience, Notthingham, UK; T-BET cDNA: IRATp970D0558D; EOMES cDNA: IRAKp961A1269Q) to the 5’ end of the mutated human estrogen receptor ligand-binding domain ER^T2^ (21). The T-BET- and EOMES-ER^T2^ constructs were ligated, separately, into the LZRS-P2A-eGFP retroviral vector. The LZRS-ER^T2^-P2A-eGFP vector, lacking T-BET or EOMES cDNA, was used as negative control. Retrovirus was produced as previously described (22).

### 2.2 HPC isolation from umbilical cord blood

Umbilical cord blood (UCB) was obtained from the Cord Blood Bank of Ghent University (Ghent University Hospital, Ghent, Belgium). Cord blood usage was approved by the Ethics Committee of the Faculty of Medicine and Health Sciences of Ghent University. Informed consents were received according to the Declaration of Helsinki. Mononuclear cells were isolated using Lymphoprep density gradient centrifugation and CD34^+^ hematopoietic progenitors cells (HPCs) were subsequently enriched with the direct CD34^+^ HSC Microbead kit (Miltenyi Biotech, Leiden, The Netherlands).

### 2.3 Generation of mature ILC3 from stem cell precursors

UCB-derived CD34^+^ HPCs were cultured for 48 h in complete Icove’s Modified Dulbecco’s Medium (Thermo Fisher Scientific, Waltham, MA) containing 10% Fetal Calf Serum (FCS; Biowest, Nuaillé, France), 100 U/ml penicillin, 100 µg/ml streptomycin and 2mM glutamine (all from Thermo Fisher Scientific) and supplemented with 20 ng/ml thrombopoietin (TPO), 100 ng/ml stem cell factor (SCF) (Peprotech, London, UK) and 100 ng/ml FMS-like tyrosine kinase 3 ligand (FLT3-L) (R&D Systems, Minneapolis, MN). Thereafter, these cells were transferred to RetroNectin (12 µg/ml)-coated plates and transduced with retroviral supernatant by spinning at 950 g for 90 minutes at 32°C. After 48 h of transduction, CD34^+^Lin(CD3, CD14, CD19, CD56)^-^eGFP^+^ HPCs were sorted using a BD FACSAria™ Fusion cell sorter (BD Biosciences, San Jose, CA) and were cultured on inactivated EL08.1D2 feeder cells (23, 24) in differentiation medium consisting of Dulbecco’s modified Eagle medium and Ham’s F-12 medium (2:1 ratio) (all from Thermo Fisher Scientific), supplemented with 100 U/mL penicillin, 100 μg/mL streptomycin, 2 mM glutamine, 10 mM sodium pyruvate (Thermo Fisher Scientific), 20% heat-inactivated human AB serum (Biowest), 24 μM β-mercaptoethanol, 20 μg/mL ascorbic acid and 50 ng/mL sodium selenite (all from Sigma-Aldrich, Saint-Louis, MO). To promote ILC3 differentiation, IL-3 (5 ng/mL, R&D systems), IL-7 (20 ng/mL), IL-15 (10 ng/mL) (Miltenyi Biotec), SCF (20 ng/mL), and Flt3-L (10 ng/mL) were added to the culture medium. On day 7 the culture medium was refreshed by doubling the medium supplemented with the above mentioned cytokines, excluding IL-3. Between day 12 and 14, depending on cell density, cells were harvested and transferred to new inactivated feeder cells in fresh medium containing cytokines.

### 2.4 ILC3-NK cell transdifferentiation cultures

Following an 18 day culture period, eGFP^+^CD45^+^CD34^-^CD11a^-^CD94^-^CD56^+^CD117^+^NKp44^+^ mature ILC3 were sorted with the BD FACSAria III cell sorter (BD Biosciences). The sorted ILC3, containing the T-BET/EOMES fusion protein or the control construct, were then cultured in 48 well plates without feeder cells at cell density of 160,000 cells/ml in the same differentiation medium containing IL-7, IL-15, SCF and Flt3-L, as described above, and in the presence or absence of 4-Hydroxytamoxifen (4-OHT, 900 nM, Sigma Aldrich). The cultures were replenished with 4-OHT every 24 h. After 7 days the medium was refreshed and cells were cultured up to 14 days.

Following a 7-day culture period, eGFP^+^CD94^-^CD117^-/low^CD56^+^ cells from T-BET overexpression cultures were sorted with the BD FACSAria III cell sorter (BD Biosciences) and replated for an additional 7-day culture period in the same differentiation medium containing IL-7, IL-15, SCF and FLT3-L, as described above and in the presence of 4-OHT (900nM, Sigma Aldrich). The cultures were replenished with 4-OHT every 24 h.

### 2.5 Flow cytometry

Utilized antibodies are listed in **Supplementary Table 1**. To perform intracellular and intranuclear analysis, the BD Cytofix/Cytoperm kit (BD Biosciences) and the FoxP3/Transcription Factor Staining buffer set (Thermo Fisher Scientific) were used, respectively, according to the manufacturer’s instructions. Cells were acquired using the LSRII flow cytometer (BD Biosciences) and data were analyzed with FlowJo_V10.8.1 software (Ashland, OR).

### 2.6 Functional assays

#### 2.6.1 Cytokine stimulation

To induce IFN-γ production, cells from day 14 T-BET/EOMES-ER^T2^ overexpression and control cultures were stimulated with IL-12, IL-18 and IL-15 (all 10 ng/ml) for 24 h. To induce IL-22 production, cells were stimulated with IL-1β and IL-23 (both 50 ng/ml) for 24 h. Phorbol myristate acetate (PMA; 50 ng/ml) and ionomycin (1 µg/ml) were added 6 hours prior to harvesting. During the 24 h stimulation, cells from 4-OHT cultures were additionally supplemented with 4-OHT. Brefeldin A (BD GolgiPlug) was added during the final 4 h of stimulation. Intracellular IFN-γ and IL-22 expression was analyzed by flow cytometry. To measure cytokine secretion, the above mentioned cells were exposed to the previously described cytokines for 24 h in the absence of Brefeldin, whereafter supernatant was collected and an IFN-γ (PeliKine compact human IFN-γ kit; Sanquin, Amsterdam, The Nederlands) or IL-22 ELISA assay (DuoSet ELISA for human IL-22; R&D systems) was performed.

#### 2.6.2 Degranulation assay

Day 14 control, T-BET- and EOMES-ER^T2^ overexpression cells were co-cultured with K562 target cells at a 1:1 ratio for 2 h. Co-cultures without K562 cells were the controls for spontaneous degranulation. Thereafter, CD107a expression on gated NK cells was determined by flow cytometry. CD107a percentages of spontaneous degranulation were subtracted from the percentages obtained with K562 stimulation.

#### 2.6.3 ^51^Chromium release assay

K562 target cells were labelled with Na_2_^51^CrO_4_ (Perkin Elmer, Waltham, MA) and challenged with bulk control, T-BET- and EOMES-ER^T2^ overexpression cells at variable effector to target ratio’s with or without 4-OHT. As controls, medium or 2% Triton X-100 were added instead of effector cells to obtain the spontaneous and total ^51^Cr release respectively. After 4 h of incubation, supernatant was harvested and mixed with scintillation fluid. The signal was measured using a 1450 LSC&Luminiscence Counter (Wallac Microbeta Trilux, Perkin Elmer). Specific cell lysis was calculated with the following formula: 100 x [(experimental release - spontaneous release)/(total release - spontaneous release)].

### 2.7 Statistical analysis

Statistical significance was determined with multiple paired t-tests (Holm-Šídák correction for multiple comparisons) and Two-Way ANOVA using GraphPad Prism version 9.3.1 (San Diego, CA). P-values< 0.05 were considered statistically significant.

## 3 Results

### 3.1 Inducible T-BET and EOMES overexpression in mature ILC3 results in the generation of NK cells upon ectopic T-BET expression only

**Figure 1:**
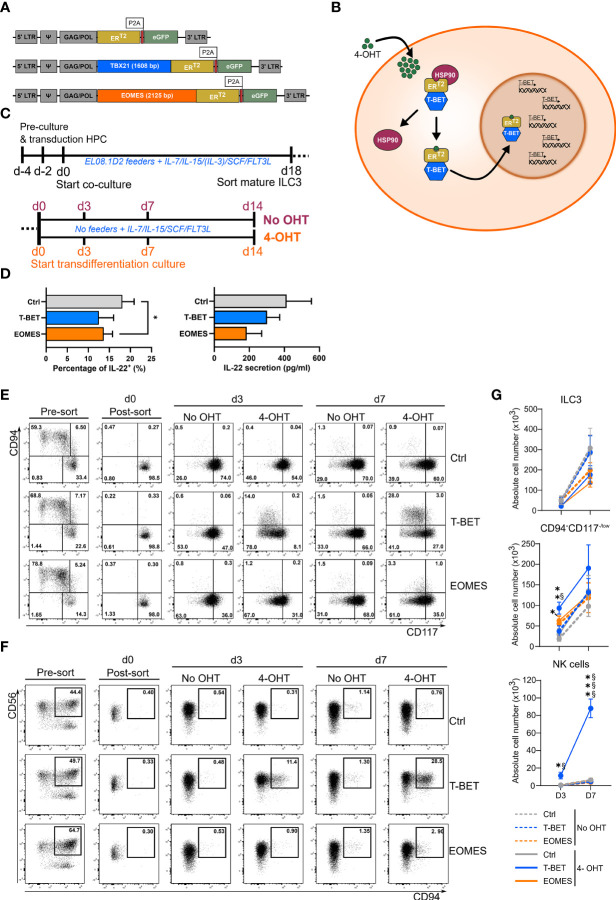
Inducible T-BET and EOMES overexpression in human HPC-derived mature ILC3s results in the generation of NK cells upon ectopic T-BET expression only. **(A, B)** Schematic representation of the inducible retroviral overexpression system of T-BET, EOMES and control conditions. **(A)** Viral construct composition. **(B)** Mechanism of action in which the T-BET inducible overexpression construct is set as an example for both transcription factors. Transcription of the retroviral construct results in a fusion protein of the transcription factor and the mutated human estrogen receptor ligand-binding domain ER^T2^, which has a high affinity for Tamoxifen. This fusion protein is retained in the cytoplasm, in an inactive state, by binding to heat shock proteins (HSP90). When 4-Hydroxytamoxifen (4-OHT) is added, it binds to the ER^T2^ receptor causing the fusion protein to detach from HSP90 and to translocate into the nucleus. Once inside the nucleus, the transcription factor is active and exerts its regulatory functions. **(C)** Schematic overview of the experimental outlines. First, HPC are transduced with the inducible retroviral constructs and differentiated towards mature ILC3s whereafter mature ILC3s (eGFP^+^CD45^+^CD34^-^CD94^-^CD11a^-^CD56^+^CD117^+^NKp44^+^) are sorted (top). Sorted ILC3s are cultured under feeder-free culture conditions in the presence or absence of 4-OHT and analyzed at the indicate time periods (bottom). **(D)** Mature ILC3s (eGFP^+^CD94^-^CD117^+^NKp44^+^) on day 18 of the ILC3 differentiation culture were stimulated with a combination of IL1β and IL-23 in addition to PMA/Ionomycin for 24 h. Percentages of IL-22^+^ cells (left) and the concentration of secreted IL-22 (pg/ml) (right) are shown (mean ± SEM; n = 3-4). **(E–G)** T-BET, EOMES and control transduced mature ILC3 (eGFP^+^CD45^+^CD34^-^CD94^-^CD11a^-^CD56^+^CD117^+^NKp44^+^) were sorted on day 18 of the ILC3 differentiation culture. The mature ILC3s were then cultured in the presence or absence of 4-OHT. **(E, F)** Representative dot plots of eGFP^+^ -gated cells pre- and post-sort and at the indicated time points of culture in the absence or presence of 4-OHT. The numbers in the plots indicate the percentages. **(E)** CD94 versus CD117 staining. The sorted ILC3s are CD94^-^CD117^+^ (lower right quadrant). **(F)** CD56 versus CD94 staining; the indicated gate contains the CD56^+^CD94^+^ NK cells. **(G)** Absolute cell numbers of ILC3s (CD94^-^CD117^+^NKp44^+^RORγt^+^), CD94^-^CD117^-/low^CD56^+^ cells and NK cells (CD56^+^CD94^+^) at the indicated time points post-sort (mean ± SEM; n = 4-11). Multiple paired t-tests with Holm-Šídák correction were used to confirm statistical significance. *, **, *** indicate significant difference of the indicated overexpression condition compared to the control, with p<0.05, p<0.01, p<0.001, respectively. Statistical difference of no OHT vs. 4-OHT conditions is indicated as § and §§§, with p<0.05 and p<0.001, respectively.

A human RORγt^+^ ILC precursor has been defined that gives rise to both NK cells and ILC3s (20)*,* indicating that these populations are more closely related than originally thought. This also suggests that there might be some degree of plasticity between ILC3s and NK cells. As T-BET and EOMES are crucial transcription factors in NK cell differentiation, we examined whether ectopic overexpression of either T-BET or EOMES in human ILC3s can transform them into NK cells. T-BET and EOMES were cloned, separately, into an inducible vector construct, creating a fusion protein of the transcription factor and the human estrogen receptor ligand-binding domain ER^T2^. The control construct contained the ER^T2^ receptor only (**Figure 1A**). The fusion protein is retained in the cytoplasm in an inactive state by binding to heat shock proteins (HSP90). When 4-Hydroxytamoxifen (4-OHT) is added, this binds to the ER^T2^ receptor causing the fusion protein to detach from HSP90 and to translocate into the nucleus. In the nucleus the transcription factor becomes active and exerts its regulatory functions (**Figure 1B**). The inducible overexpression constructs were first transduced into UCB-derived CD34^+^ HPCs, which were then differentiated towards mature ILC3s. On day 18 of culture, mature ILC3s (eGFP^+^CD45^+^CD34^-^CD11a^-^CD94^-^CD56^+^CD117^+^NKp44^+^) were sorted (**Supplemental Figure 1A**). The sorted ILC3s containing the control construct or the T-BET/EOMES fusion protein were then cultured in the presence or absence of 4-OHT (**Figure 1C**). To confirm that the sorted day 18 ILC3s were indeed mature and functional, these cells were stimulated with a combination of IL-1β and IL-23 in addition to PMA/ionomycin to evaluate their capacity to produce and secrete IL-22, which is an important functional feature of mature ILC3s. Flow cytometric analysis and evaluation by ELISA confirmed that day 18 ILC3s of all conditions were able to produce and secrete significant amounts of IL-22. T-BET- and EOMES-overexpressing ILC3s showed reduced percentages and concentrations of IL-22 compared to control ILC3s, indicating that there is some degree of leakiness of the construct (**Figure 1D**). To evaluate the NK cell generation potential upon ectopic T-BET or EOMEs expression in ILC3s, flow cytometric analysis of these populations was performed. Sorted mature ILC3s were characterized as CD94^-^CD117^+^NKp44^+^ cells, whereas NK cells were defined as CD56^+^CD94^+^ cells (**Supplemental Figure 1B**). Analysis of CD94 and CD117 expression on days 3 and 7 revealed that in the absence of 4-OHT all conditions acted similar. The majority of the cells remained CD94^-^CD117^+^, although a considerable amount of cells lost CD117 expression and became CD94^-^CD117^-/low^ (**Figures 1E, G**). With the addition of 4-OHT, both the percentage as well as the absolute cell number of CD94^-^CD117^-/low^CD56^+^ cells of T-BET and EOMES overexpression cultures increased on day 3 compared to control cultures and continued to increase towards day 7 (**Figures 1E–G**). However, ectopic T-BET expression resulted in a drastic increase of both percentages and absolute cell numbers of CD94^+^ NK cells on day 3, compared to control cultures, which became even more prominent on day 7. In contrast, EOMES overexpression in ILC3s resulted in low amounts of CD94^+^ NK cells, that were not significantly different from control cultures (**Figures 1F, G**). Altogether, ectopic T-BET expression in mature ILC3s results in transdifferentiation towards CD94^+^ NK cells, whereas ectopic EOMES expression in ILC3s does result in dedifferentiation into CD94^-^CD117^-/low^CD56^+^cells, but is ineffective in generating CD94^+^ NK cells.

### 3.2 Transdifferentiation of ILC3s into NK cells occurs only upon high ectopic T-BET expression and subsequent downregulation of RORγt expression

**Figure 2:**
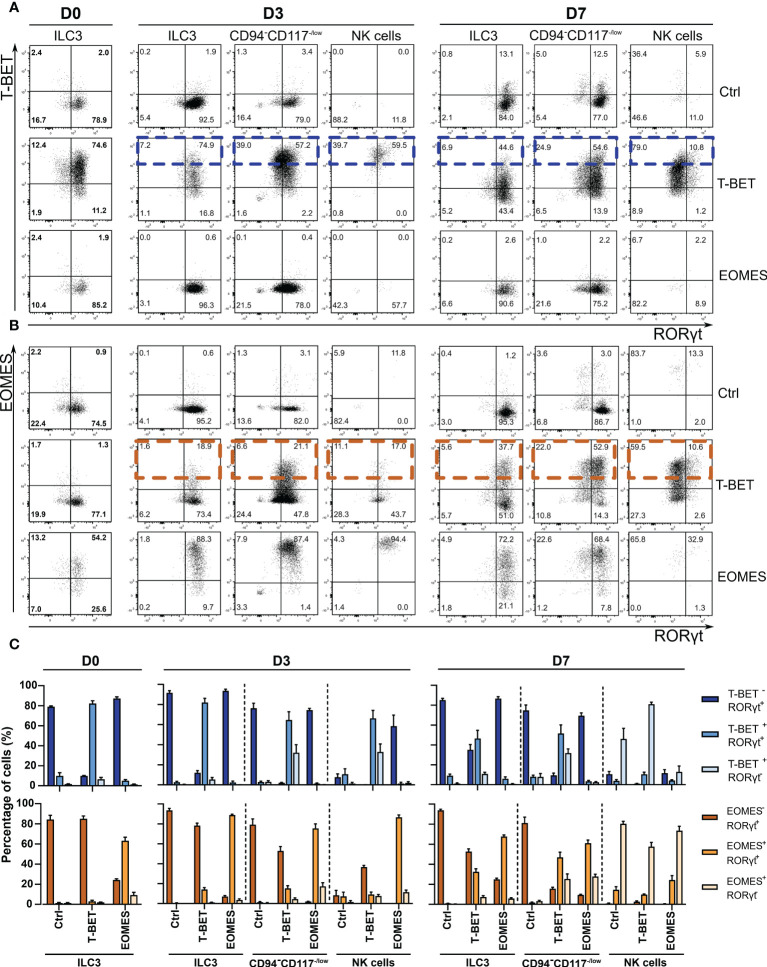
T-BET, EOMES and RORγt expression during ILC3-NK cell transdifferentiation culture in the presence of 4-OHT. Sorted ILC3s overexpressing T-BET and EOMES and control ILC3s were cultured in the presence or absence of 4-OHT. Transcription factor analysis was performed on the indicated time points by flow cytometry on gated eGFP^+^ cells. **(A, B)** Representative dot plots of the indicated populations showing bimodal transcription factor expression on the indicated time points during culture in the presence of 4-OHT only. **(A)** T-BET vs. RORγt staining. High T-BET expressing cells in the T-BET -overexpressing condition are highlighted with blue rectangles. **(B)** EOMES vs. RORγt staining. Orange rectangles indicate high EOMES -expressing cells in the T-BET -overexpressing condition. The numbers in the plots indicate the percentages. **(C)** Frequencies of bimodal transcription factor expression for the different time points and different populations as indicated (mean ± SEM; n = 4).

Approximately, 28.5% of eGFP^+^ cells on day 7 was able to transdifferentiate into NK cells upon ectopic T-BET expression in ILC3s (**Figure 1F**). This indicates that the large majority of the transduced ILC3s fails to upregulate CD94 to become NK cells. To further address this finding, analysis of the transcription factor patterns in the different populations was performed by flow cytometry. At day 0 of culture, T-BET and EOMES overexpression in sorted ILC3s was confirmed at the protein level (**Figures 2A–C**). Both T-BET and EOMES overexpression conditions show a clear bimodal expression of T-BET or EOMES, respectively, and of RORγt, wherein RORγt is the signature transcription factor driving ILC3 development and differentiation. Accordingly, high RORγt expression was observed in the sorted ILC3s on day 0 (**Figures 2A–C** and **Supplemental Figures 2A–C**) and this expression was maintained throughout the different cultures in the absence of 4-OHT (**Supplemental Figures 2A–C**). However, upon addition of 4-OHT, ectopic T-BET expression gradually increased from ILC3s towards NK cells in the T-BET cultures on days 3 and 7, wherein ILCs had the lowest T-BET expression levels (**Figure 2A**, blue rectangles). Moreover, T-BET overexpression was further upregulated after the loss of CD117 in the CD94^-^CD117^-/low^CD56^+^ cells and NK cells expressed the highest levels of T-BET on both days 3 and 7. In contrast, EOMES expression was already high in ILC3s upon ectopic EOMES expression and this expression level was maintained throughout the culture (**Figures 2A–B**). Bimodal expression analysis revealed that in T-BET overexpression cultures 51.6% ± 8.5% and 31.8% ± 4.2% of the CD94^-^CD117^-/low^CD56^+^ cells were T-BET^+^RORγt^+^ and T-BET^+^RORγt^-^, respectively, while 81% ± 2.0% of the NK cells were T-BET^+^ RORγt^-^. However, the same was partially true for EOMES overexpression cultures as 61.0% ± 3.0% and 27.7% ± 2.4% of the CD94^-^CD117^-/low^CD56^+^ cells were EOMES^+^RORγt^+^ and EOMES^+^RORγt^-^, respectively (**Figure 2C**). This indicates that RORγt expression levels were downregulated upon high T-BET or EOMES expression, which implies the occurrence of a cell-identify shift. Despite the fact that EOMES similarly reduced RORγt expression in the CD94^-^CD117^-/low^CD56^+^ population on day 7 compared to T-BET overexpression, ectopic EOMES expression in ILC3s resulted in low amounts of CD94^+^ NK cells that were not significantly different from control cultures (**Figures 1F, G**). In the absence of 4-OHT, as expected, T-BET overexpression did not result in endogenous EOMES expression and vice versa (**Figures 2A–C** and **Supplemental Figures 2A–C**). However, in the presence of 4-OHT, ectopic T-BET expression in ILC3s induced EOMES expression from day 3 onwards (**Figures 2B, C**, orange rectangles). In contrast, ectopic EOMES expression did not result in endogenous T-BET expression at any of the time points examined. The NK cells generated upon overexpression of T-BET upregulated EOMES, but to a lesser extent as in control NK cells. Strikingly, the few NK cells originating from EOMES-overexpressing ILC3s completely lacked expression of T-BET (**Figures 2A–C**). In conclusion, upon ectopic T-BET expression the majority of the ILC3s express intermediate levels of T-BET and remain bona fide ILC3s. However, a proportion of these cells express high T-BET levels, downregulate RORγt expression and eventually become CD94^+^ NK cells. This indicates that only ILC3s with high T-BET expression levels will transdifferentiate into NK cells and additionally induce EOMES expression. In contrast, high EOMES expression downregulates RORγt, but fails to induce T-BET expression resulting in an almost complete absence of NK cells.

### 3.3 ILC3s transforming into NK cells gradually express NK cell characteristics by which transdifferentiation does not necessarily occur *via* a CD94^-^CD117^-/low^CD56^+^ stadium

**Figure 3:**
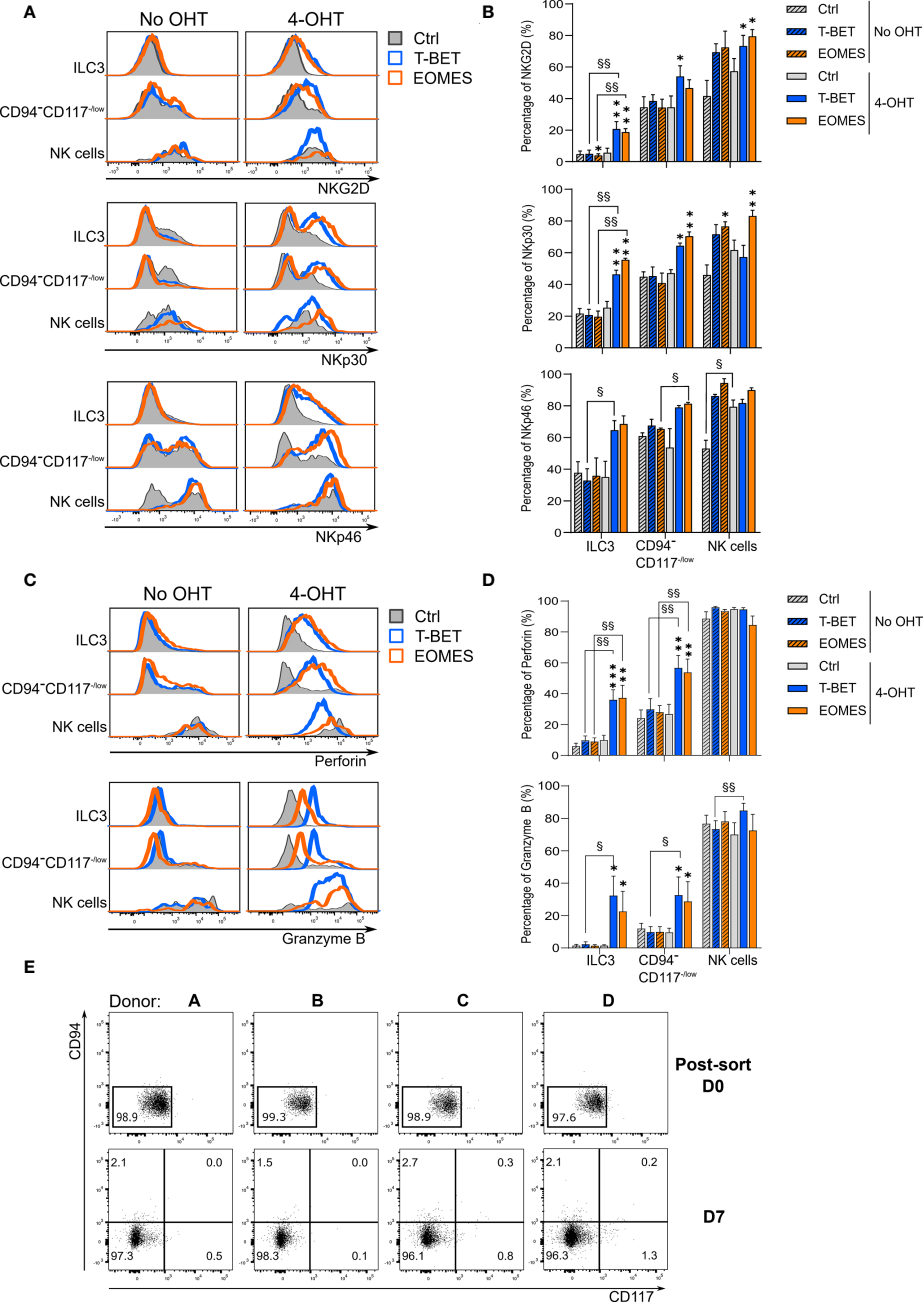
ILC3s transdifferentiating into NK cells gradually express NK cell characteristics by which transdifferentiation does not necessarily occurs *via* a CD94^-^CD117^low^CD56^+^ stadium. ILC3s overexpressing T-BET or EOMES and control ILC3s are cultured in the presence or absence of 4-OHT. Analysis was performed on day 7 by flow cytometry and eGFP^+^ cells were gated. **(A)** Representative histograms of NKG2D, NKp30 and NKp46 expression in the populations as specified. **(B)** Percentages of NKG2D, NKp30 and NKp46 expression (mean ± SEM; n = 2-6). **(C)** Representative histograms of perforin and granzyme B expression in the populations as specified. **(D)** Percentages of perforin and granzyme B expression (mean ± SEM; n = 6). Multiple paired t-tests with Holm-Šídák correction were used to confirm statistical significance. Significant difference of the indicate overexpression condition vs. control is shown as *, ** and ***, with p < 0.05, p < 0.01 and p < 0.001, respectively. Statistical difference of no OHT vs. 4-OHT conditions is indicated as § and §§, with p < 0.05 and p < 0.01. **(E)** eGFP^+^ CD94^-^CD117^-/low^CD56^+^ cells were resorted and replated on day 7 of the ILC3-NK cell transdifferentiation culture. Analysis of the NK cell generation potential was performed by flow cytometry after an additional 7-day culture. Depicted are the dot plots of CD94 vs. CD117 expression of the 4 donors on the time points as specified. The numbers indicate the percentages.

To further characterize the transdifferentiation of ILC3s *via* the CD94^-^CD117^-/low^CD56^+^ population into CD94^+^ NK cells, the phenotype of the different populations was determined by flow cytometry. On day 7 of the transdifferentiation culture, 4-OHT treated ILC3s with ectopic T-BET and EOMES overexpression expressed a significant higher percentage of the activating NK cell receptors NKG2D, NKp30 and NKp46 compared to control conditions with and without 4-OHT (**Figures 3A, B**). Furthermore, the expression of these activating NK cell receptors was gradually upregulated in the CD94^-^CD117^-/low^CD56^+^ population with T-BET and EOMES induced overexpression, wherein the percentages were still higher compared to the control conditions (**Figures 3A, B**). The resulting NK cells of the induced T-BET and EOMES cultures had similar NKp46 expression compared to all control NK cells. NKG2D expression in these NK cells is significantly higher with both ectopic T-BET and EOMES expression, while NKp30 was significantly higher expressed in NK cells from induced EOMES overexpression cultures only (**Figures 3A, B**). In contrast to the activating NK cell receptors, expression of CD16 or KIRs could not be detected in the resulting NK cells in any of the different conditions investigated, even on day 14 of culture (**Supplementary Figure 3**).

NK cells are known producers of perforin and granzyme B to elaborate their killing capacity, whereas ILC3s do not express these cytotoxic mediators. Strikingly, ILC3s from both induced T-BET and EOMES overexpression cultures clearly expressed perforin and granzyme B. As expected, control ILC3s were negative for both cytotoxic effector proteins (**Figures 3C, D**). The same was true for the CD94^-^CD117^-/low^CD56^+^ population, wherein both the perforin and granzyme B expression level substantially increased upon induced T-BET and EOMES overexpression (**Figures 3C, D**). The resulting NK cells from induced T-BET and EOMES expression cultures had perforin and granzyme B levels that were comparable to control NK cells, whereas granzyme B expression was slightly, but significantly higher upon ectopic T-BET overexpression compared to the T-BET culture without 4-OHT. (**Figures 3C, D**). Analysis of the transcription factor expression patterns (**Figure 2**) and phenotypical analysis (**Figure 3C, D**) of the CD94^-^CD117^-/low^CD56^+^ cells indicated that these cells might be an intermediate population in the ILC3-NK cell transdifferentiation pathway. To test this hypothesis, eGFP^+^ CD94^-^CD117^-/low^CD56^+^ cells from the T-BET overexpression conditions in the presence of 4-OHT were sorted on day 7 (**Supplemental Figure 4**) and subsequently cultured for an additional 7 days in the ILC3-NK cell transdifferentiation culture system. Flow cytometric analysis revealed that the CD94^-^CD117^-/low^CD56^+^ cells retained their phenotype and did not differentiate into CD94^+^ NK cells (**Figure 3E**).

Thus, both T-BET and EOMES ectopic expression induces expression of activating NK cells receptors as well as perforin and granzyme B in ILC3s and in the CD94^-^CD117^-/low^CD56^+^ cells. However, despite their intermediate phenotype, isolated CD94^-^CD117^-/low^CD56^+^cells from T-BET overexpression conditions fail to generate NK cells. As a consequence, CD94^-^CD117^-/low^CD56^+^ cells are not a mandatory intermediate stage of ILC3-NK cell transdifferentiation.

### 3.4 Transdifferentiating ILC3s lose the capability to produce IL-22 and gain the capacity to produce IFN-γ and become cytotoxic

**Figure 4:**
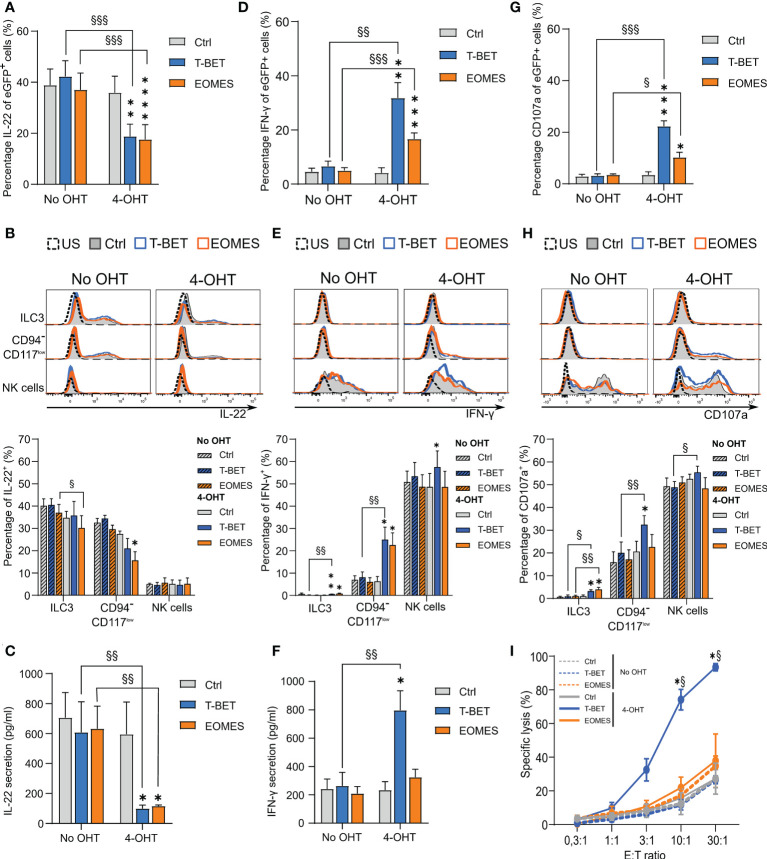
Transdifferentiating ILC3s lose the capability to produce IL-22 and gain the capacity to produce IFN-γ and become cytotoxic. Sorted ILC3s from T-BET, EOMES and control conditions were cultured in the presence or absence of 4-OHT for 14 days and then subjected to different functionality assays. **(A, B)** Intracellular IL-22 production after 24 h of stimulation with a combination of IL1β and IL-23 in addition to PMA/Ionomycin **(A)** The percentages of IL-22^+^ cells in gated eGFP^+^ cells (mean ± SEM; n = 5). **(B)** Representative histograms (upper part) and the percentages of IL-22^+^ cells (lower part) in the indicated populations are shown (mean ± SEM; n = 5). **(C)** The amount of IL-22 secreted by cells from the specified conditions as measured by ELISA after 24 h of stimulation with cytokines as mentioned in A. **(D, E)** Intracellular IFN-γ production after 24 h of stimulation with a combination of IL-12, IL-18 and IL-15. **(D)** The percentages of gated eGFP^+^ cells expressing IFN-γ (mean ± SEM; n = 6). **(E)** Representative histograms (upper part) and bar charts (lower part) demonstrating the percentage of IFN-γ^+^ cells in the indicated populations (mean ± SEM; n = 6). **(F)** The amount of secreted IFN-γ by cells from the specified conditions as determined by ELISA after 24 h stimulation with cytokines as mentioned in C. **(G, H)** Cells were challenged with K562 cells during a 2 h CD107a degranulation assay. **(G)** The percentages of CD107a -expressing cells in the gated eGFP^+^ population (mean ± SEM; n = 6). **(H)** Representative histograms (upper part) and the percentage of CD107a^+^ cells (lower part) of the indicated populations are shown (mean ± SEM; n = 6). **(B, E, H)** US = unstimulated condition. **(I)** Cells were challenged with K562 cells at different effector over target (E:T) ratio’s in a ^51^Chromium release assay. The percentage of the specific target cell lysis is shown (mean ± SEM; n = 3). **(A-H)** Multiple paired t-tests with Holm-Šídák correction were used to confirm statistical significance. **(I)** Two-way ANOVA was performed to confirm statistical significance. **(A-I)** Significant difference of the indicate overexpression condition vs. control is shown as *, **, *** and **** with p<0.05, p<0.01, p<0.001 and p <0.0001, respectively. Statistical difference of no OHT vs. 4-OHT conditions is indicated as §, §§ and §§§ with p < 0.05, p < 0.01 and p < 0.001, respectively. .

ILC3s are known producers of IL-22, while NK cells are important IFN-γ producers. To examine the cytokine expression and secretion profile of these populations, cells of the different conditions were stimulated with cytokines on day 14 of the transdifferentiation culture. Flow cytometric analysis upon stimulation with a combination of IL-1β and IL-23 in addition to PMA/ionomycin showed that upon ectopic T-BET or EOMES expression the total eGFP^+^-gated cells displayed a drastic reduction in IL-22 production (**Figure 4A**). Detailed analysis of the different subpopulations revealed that ILC3s of all conditions indeed produced IL-22, wherein IL-22 production in EOMES-overexpressing ILC3s in the presence of 4-OHT was significantly lower compared to the condition without 4-OHT. The CD94^-^CD117^low^CD56^+^ cells further downregulated IL-22 expression predominantly with ectopic EOMES expression. As expected, the resulting NK cells from all conditions showed little to no IL-22 expression (**Figure 4B**). Assessment of secretion of IL-22 with ELISA in stimulated bulk cells from day 14 cultures showed that T-BET- as well as EOMES-overexpressing cells in the presence of 4-OHT had a drastically reduced capacity to secrete IL-22 in comparison to the control conditions (**Figure 4C**). Almost the exact opposite was true for IFN-γ production. Stimulation with a combination of IL-12, IL-15 and IL-18 resulted in a strong increase in IFN-γ production in the total eGFP^+^-gated cells from T-BET and EOMES cultures after addition of 4-OHT, wherein T-BET was more efficient (**Figure 4D**). Evaluation of the IFN-γ production in the different subpopulations revealed that little to no IFN-γ production was induced in ILC3s of all conditions. However, IFN-γ expression was significantly increased in the T-BET- and EOMES-overexpressing CD94^-^CD117^-/low^CD56^+^ cells in comparison to cells from the control culture. The NK cells displayed a similar percentage of IFN-γ expression in all conditions, although NK cells arising in the ectopic T-BET expression cultures expressed a significantly higher percentage of IFN-γ (**Figure 4E**). Evaluation of IFN-γ secretion as determined by ELISA in stimulated bulk cells from day 14 cultures revealed that T-BET-overexpressing cells in the presence of 4-OHT produced drastically more IFN-γ compared to EOMES-overexpressing cells and the control conditions (**Figure 4F**).

NK cells do not only produce cytokines, they are also cytotoxic. To evaluate the cytotoxic potential of NK cells generated from transdifferentiated ILC3s, a CD107a degranulation assay was performed. Co-culture of cells from day 14 cultures with K562 target cells revealed that total eGFP^+^-gated cells from T-BET and EOMES cultures displayed higher CD107a expression compared to cells from control cultures, wherein T-BET was dominant in upregulating CD107a expression (**Figure 4G**). Detailed analysis of the different subpopulations revealed that NK cells within all conditions displayed a high percentage of CD107a expression on the cell surface, wherein NK cells differentiated from T-BET -overexpressing ILC3s in the presence of 4-OHT showed significantly higher CD107a levels. The stronger CD107a upregulation in induced T-BET overexpression cultures became even more clear in the CD94^-^CD117^-/low^CD56^+^ cell population. Finally, also the remaining ILC3s in both induced T-BET and EOMES overexpression cultures showed a low, but significant upregulation of CD107a compared to the control conditions (**Figure 4H**). As CD107a expression is a measure of degranulation of cytotoxic granules, it does not provide formal evidence of cytotoxicity. Therefore, a ^51^Cr release assay with day 14 culture cells was performed with K562 as target cells. Compared to the cells from the control cultures, only T-BET-overexpressing cells in the presence of 4-OHT displayed a strongly increased killing efficiency (**Figure 4I**).

In conclusion, whereas both ectopic T-BET and EOMES overexpression in ILC3s completely block IL-22 secretion, only T-BET overexpression increases IFN-γ secretion as well as cytotoxicity against tumor target cells.

## 4 Discussion

To investigate ILC3-NK cell plasticity we ectopically overexpressed either T-BET or EOMES in human, mature ILC3s. T-BET and EOMES are key transcription factors in human NK cell differentiation. Human NK cells are characterized by a T-BET and EOMES gradient, wherein T-BET and EOMES have a reciprocal relationship. As NK cells mature they upregulate T-BET expression and downregulate EOMES expression, whereby T-BET^high^EOMES^low^ cells are considered as terminally mature NK cells (25, 26). Recently, it has been demonstrated that both in mouse and human EOMES plays a dominant role in early NK cell maturation, whereas T-BET is more efficient in inducing terminal NK cell maturation. This implicates that EOMES acts upstream of T-BET in the NK cell maturation cascade (24, 27). Our results demonstrate that only upon high ectopic T-BET expression ILC3s can upregulated CD94 and ultimately converted into CD94^+^ NK cells. In contrast, 4-OHT-mediated activation of EOMES expression in mature ILC3s resulted in dedifferentiation into the CD94^-^CD117^-/low^ cells, but did not yield a significant number of cytotoxic NK cells. More surprisingly, ectopic EOMES expression also downregulated RORγt expression in CD94^-^CD117^-/low^ cells. Evaluation of the T-BET and EOMES expression pattern showed that T-BET overexpression in the presence of 4-OHT upregulated EOMES expression in ILC3s, ultimately leading to transformation into NK cells. In contrast, ectopic EOMES overexpression in the presence of 4-OHT did not induce endogenous T-BET expression in ILC3s. The observation that EOMES is an early regulator of the NK cell fate (24, 27) suggests that overexpression of EOMES alone is not sufficient to generate significant amounts of NK cells from ILC3s, as T-BET is required for further NK cell differentiation and subsequent proliferation (24, 27) The low number of differentiated NK cells from EOMES overexpressing ILC3s expressed NK cell receptors as well as cytotoxic effector molecules, as is also the case for early differentiated NK cells (24, 27).

Ectopic T-BET and EOMES expression in human ILC3s resulted in downregulation of RORγt and loss of CD117 expression, whereby these cells eventually upregulated CD94 expression with ectopic T-BET expression only. Previously, intermediate innate subsets that can differentiate into both ILC3s and NK cells have been identified in human fetal intestine and they can be distinguished by differential expression of CD94 and CD117. The CD94^-^CD117^-^ subset could be positioned between CD94^+^CD117^-^ and CD94^-^CD117^+^ subsets on the basis of transcription factor expression analysis, wherein the CD94^-^CD117^-^ subset only partially expresses RORγt (28). Characterization of the CD94^-^CD117^-/low^CD56^+^ cells here, revealed that they expressed NKG2D, NKp30, NKp46, perforin and granzyme B at higher levels than ILC3s, but still lower compared to CD94^+^ NK cells. The CD94^-^CD117^-/low^CD56^+^ cells also displayed an intermediate level of RORγt expression, similar as observed in the above mentioned intermediate ILC subset (28). Despite their intermediate phenotypical pattern, isolated CD94^-^CD117^-/low^CD56^+^ cells from the T-BET-overexpressing conditions did not convert into CD94^+^ NK cells, which implicates that this population is not a mandatory intermediate stage in ILC3-NK cell transdifferentiation, at least *in vitro*.

ILC3 to ILC1 transdifferentiation and their intermediate stages are well documented in both mice and human (8–13, 20, 28). ILC3 to NK cell transdifferentiation in humans has also been reported (29, 30), but is less well documented. Nevertheless, a clear distinction between human ILC1 and NK cells remains challenging as their phenotype and function are largely overlapping (2, 31). The CD94^+^ cells differentiated from ILC3s in this study expressed CD56, NKp44, NKp46 and CD94, which are generally not expressed on ILC1s (2, 32). Besides, these CD94^+^ cells also expressed perforin and granzyme B and were highly cytotoxic. ILC1s, on the contrary, do not express cytotoxic effector molecules and are generally non-cytotoxic (2, 31). T-BET is typically expressed by both human ILC1s and NK cells, whereas EOMES expression is restricted to NK cells (2, 13, 28, 31). CD94^+^ NK cells resulting from ectopic T-BET expression cultures additionally expressed EOMES, although at lower levels than in NK cells from control cultures. Decreased EOMES expression in these cells can be attributed to T-BET overexpression as there is a delicate balance between T-BET and EOMES expression in NK cells (24, 26, 27, 33). Thus, the CD94^+^ cells transdifferentiated from mature ILC3s in this study are not ILC1s, but are characterized as genuine NK cells.

The transdifferentiating ILC3s with ectopic T-BET or EOMES expression lost typical ILC3 features, like their ability to produce IL-22. The CD94^+^ NK cells differentiated from mature ILC3s overexpressing T-BET acquired typical NK cell receptors and were functional both in terms of IFN-γ production and cytotoxicity, but lacked expression of the mature NK cell hallmarks, CD16 and KIRs. Considering the different phenotypic and functional characteristics of the differentiated NK cells, these cells show high similarity with the NK cells described in the work of Raykova A. and colleagues (30). They have reported ILC3-NK cell plasticity *in vitro* after stimulation of *ex vivo* human tonsillar ILC3 with IL-12 and IL-15. The IL-22 producing ILC3s acquire an NK cell profile including CD94, EOMES and T-BET expression, upregulation of typical NK cell receptors and induction of cytotoxic activity (30). Here, 4-OHT activated T-BET overexpression in *in vitro* generated ILC3s mimics in some part the effect of IL-12, which is a known inducer of T-BET expression by binding IL12RB2 and activating STAT4. T-BET, on its turn, induces IL12RB2 expression rendering the cells more responsive to IL-12 (34).

The resulting CD94^+^ NK cells from this study and as reported by Raykova A. and colleagues (30) closely resemble stage 4b cells of the NK cell developmental pathway as described by Freud A.G. and colleagues (35). Stage 4b NK cells are defined as CD34^-^CD117^+/-^CD94^+^NKp80^+^CD16^-^ cells, which express NK cell receptors, produce IFN-γ and have cytotoxic capacity. In contrast to the NK cells defined in our study, KIR^+^ subsets were detected within the stage 4b population (35). KIR expression was also absent in NK cells derived from tonsillar ILC3s (30). We have previously demonstrated that constitutive ectopic T-BET expression in human HPCs followed by *in vitro* NK cell differentiation results in increased KIR expression on the generated NK cells. We therefore suggested that T-BET epigenetically regulates KIR expression (24). By chromatin accessibility profiling using Fast-ATAC sequencing, we indeed showed that KIR-specific ATAC regions are already upregulated upon constitutive T-BET overexpression in human HPCs and differentiation towards NK cells increases the accessibility of the KIR promotor regions in these ATAC sites, ultimately leading to increased KIR mRNA and protein levels (24). Here, we start from sorted ILC3s that transdifferentiate into NK cells upon T-BET activation. Our hypothesis is that ILC3s are not primed for KIR expression at the chromatin level, rendering T-BET (and EOMES) unable to induce KIR expression in these cells. However, this theory remains to be further investigated.

Recently, a cytotoxic ILC3 subset was identified in human tonsil (36). These cytotoxic ILC3s show high similarity to the NK cells obtained with 4-OHT-mediated activation of T-BET in ILC3s as they are CD94^+^ ILCs that also lack CD16 and KIR expression and additionally express CD56, NKp44, NKp46 and NKG2A. Even T-BET and EOMES are expressed, while they also express intermediate levels of RORγt. Because these cytotoxic ILC3s produce low amounts of IFN-γ and maintain the capability to produce high levels of IL-22, it is unlikely that they represent a subset of NK cells (36). Based on their capacity to produce IL-22, these cytotoxic ILC3s can be distinguished from the NK cells generated upon ILC3 transdifferentiation in this study.

In conclusion, mature ILC3s transdifferentiate into *bona fide* functional NK cells, wherein T-BET, and not EOMES, is the most potent driver.

## Data availability statement

The original contributions presented in the study are included in the article/**Supplementary material**. Further inquiries can be directed to the corresponding author.

## Ethics statement

The studies involving human participants were reviewed and approved by Ethics Committee of the Faculty of Medicine and Health Sciences of Ghent University. The patients/participants provided their written informed consent to participate in this study.

## Author contributions

Conceptualization: LK, GL. Methodology: LK, SW, EP, ZV, GL. Investigation: LK, SW, EP, ZV. Formal Analysis: LK. Visualization: LK. Supervision: GL. Writing – original draft: LK, GL. Writing – review & editing: LK, SW, EP, ZV, TT, BV, GL. All authors contributed to the article and approved the submitted version.

## Funding

This work was supported by grants from the Research Foundation – Flanders (FWO), including G.0444.17N (GL), 1S29317N (LK), 1S45317N (SW).

## Conflict of interest

The authors declare that the research was conducted in the absence of any commercial or financial relationships that could be construed as a potential conflict of interest.

## Publisher’s note

All claims expressed in this article are solely those of the authors and do not necessarily represent those of their affiliated organizations, or those of the publisher, the editors and the reviewers. Any product that may be evaluated in this article, or claim that may be made by its manufacturer, is not guaranteed or endorsed by the publisher.

## Supplementary material

Supplementary Table 1 Antibodies used for flow cytometry.

## References

[1] Spits H Artis D Colonna M Diefenbach A Di Santo JP Eberl G. Innate lymphoid cells–a proposal for uniform nomenclature. Nat Rev Immunol (2013) 13(2):145–9. doi: 10.1038/nri3365 23348417

[2] Vivier E Artis D Colonna M Diefenbach A Di Santo JP Eberl G. Innate lymphoid cells: 10 years on. Cell (2018) 174(5):1054–66. doi: 10.1016/j.cell.2018.07.017 30142344

[3] Diefenbach A Colonna M Koyasu S. Development, differentiation, and diversity of innate lymphoid cells. Immunity (2014) 41(3):354–65. doi: 10.1016/j.immuni.2014.09.005 PMC4171710 25238093

[4] Klose CSN Artis D. Innate lymphoid cells as regulators of immunity, inflammation and tissue homeostasis. Nat Immunol (2016) 17(7):765–74. doi: 10.1038/ni.3489 27328006

[5] Mazzurana L Rao A Van Acker A Mjosberg J. The roles for innate lymphoid cells in the human immune system. Semin Immunopathology (2018) 40(4):407–19. doi: 10.1007/s00281-018-0688-7 PMC6060849 29948108

[6] Colonna M. Innate lymphoid cells: Diversity, plasticity, and unique functions in immunity. Immunity (2018) 48(6):1104–17. doi: 10.1016/j.immuni.2018.05.013 PMC6344351 29924976

[7] Bal SM Golebski K Spits H. Plasticity of innate lymphoid cell subsets. Nat Rev Immunol (2020) 20(9):552–65. doi: 10.1038/s41577-020-0282-9 32107466

[8] Vonarbourg C Mortha A Bui VL Hernandez PP Kiss EA Hoyler T. Regulated expression of nuclear receptor rorgammat confers distinct functional fates to nk cell receptor-expressing rorgammat(+) innate lymphocytes. Immunity (2010) 33(5):736–51. doi: 10.1016/j.immuni.2010.10.017 PMC3042726 21093318

[9] Klose CS Kiss EA Schwierzeck V Ebert K Hoyler T d'Hargues Y. A T-bet gradient controls the fate and function of Ccr6-rorgammat+ innate lymphoid cells. Nature (2013) 494(7436):261–5. doi: 10.1038/nature11813 23334414

[10] Rankin LC Groom JR Chopin M Herold MJ Walker JA Mielke LA. The transcription factor T-bet is essential for the development of Nkp46+ innate lymphocytes *Via* the notch pathway. Nat Immunol (2013) 14(4):389–95. doi: 10.1038/ni.2545 PMC4076532 23455676

[11] Bernink JH Peters CP Munneke M te Velde AA Meijer SL Weijer K. Human type 1 innate lymphoid cells accumulate in inflamed mucosal tissues. Nat Immunol (2013) 14(3):221–9. doi: 10.1038/ni.2534 23334791

[12] Bernink JH Krabbendam L Germar K de Jong E Gronke K Kofoed-Nielsen M. Interleukin-12 and -23 control plasticity of Cd127(+) group 1 and group 3 innate lymphoid cells in the intestinal lamina propria. Immunity (2015) 43(1):146–60. doi: 10.1016/j.immuni.2015.06.019 26187413

[13] Cella M Gamini R Secca C Collins PL Zhao SR Peng V. Subsets of Ilc3-Ilc1-Like cells generate a diversity spectrum of innate lymphoid cells in human mucosal tissues. Nat Immunol (2019) 20(8):980–91. doi: 10.1038/s41590-019-0425-y PMC6685551 31209406

[14] Silver JS Kearley J Copenhaver AM Sanden C Mori M Yu L. Inflammatory triggers associated with exacerbations of copd orchestrate plasticity of group 2 innate lymphoid cells in the lungs (Vol 17, pg 626, 2016). Nat Immunol (2016) 17(8):626–35. doi: 10.1038/ni0816-1005c PMC5345745 27111143

[15] Lim AI Verrier T Vosshenrich CA Di Santo JP. Developmental options and functional plasticity of innate lymphoid cells. Curr Opin Immunol (2017) 44:61–8. doi: 10.1016/j.coi.2017.03.010 28359987

[16] Bal SM Bernink JH Nagasawa M Groot J Shikhagaie MM Golebski K. Il-1beta, il-4 and il-12 control the fate of group 2 innate lymphoid cells in human airway inflammation in the lungs. Nat Immunol (2016) 17(6):636–45. doi: 10.1038/ni.3444 27111145

[17] Cortez VS Ulland TK Cervantes-Barragan L Bando JK Robinette ML Wang QL. Smad4 impedes the conversion of nk cells into Ilc1-like cells by curtailing non-canonical tgf-beta signaling. Nat Immunol (2017) 18(9):995–1003. doi: 10.1038/ni.3809 28759002 PMC5712491

[18] Gao YL Souza-Fonseca-Guimaraes F Bald T Ng SS Young A Ngiow SF. Tumor immunoevasion by the conversion of effector nk cells into type 1 innate lymphoid cells. Nat Immunol (2017) 18(9):1004–15. doi: 10.1038/ni.3800 28759001

[19] Cuff AO Sillito F Dertschnig S Hall A Luong TV Chakraverty R. The obese liver environment mediates conversion of nk cells to a less cytotoxic Ilc1-like phenotype. Front Immunol (2019) 10:2180. doi: 10.3389/fimmu.2019.02180 31572388 PMC6749082

[20] Scoville SD Mundy-Bosse BL Zhang MH Chen L Zhang XL Keller KA. A progenitor cell expressing transcription factor ror gamma T generates all human innate lymphoid cell subsets. Immunity (2016) 44(5):1140–50. doi: 10.1016/j.immuni.2016.04.007 PMC4893782 27178467

[21] Feil R Wagner J Metzger D Chambon P. Regulation of cre recombinase activity by mutated estrogen receptor ligand-binding domains. Biochem Bioph Res Co (1997) 237(3):752–7. doi: 10.1006/bbrc.1997.7124 9299439

[22] Taghon T De Smedt M Stolz F Cnockaert M Plum J Leclercq G. Enforced expression of gata-3 severely reduces human thymic cellularity. J Immunol (2001) 167(8):4468–75. doi: 10.4049/jimmunol.167.8.4468 11591773

[23] Cichocki F Miller JS. *In vitro* development of human killer-immunoglobulin receptor-positive nk cells. Methods Mol Biol (2010) 612:15–26. doi: 10.1007/978-1-60761-362-6_2 20033631

[24] Kiekens L Van Loocke W Taveirne S Wahlen S Persyn E Van Ammel E. T-Bet and eomes accelerate and enhance functional differentiation of human natural killer cells. Front Immunol (2021) 12:732511. doi: 10.3389/fimmu.2021.732511 34630413 PMC8497824

[25] Knox JJ Cosma GL Betts MR McLane LM. Characterization of T-bet and eomes in peripheral human immune cells. Front Immunol (2014) 5:217. doi: 10.3389/fimmu.2014.00217 24860576 PMC4030168

[26] Collins A Rothman N Liu K Reiner SL. Eomesodermin and T-bet mark developmentally distinct human natural killer cells. JCI Insight (2017) 2(5):e90063. doi: 10.1172/jci.insight.90063 28289707 PMC5333970

[27] Zhang J Le Gras S Pouxvielh K Faure F Fallone L Kern N. Sequential actions of eomes and T-bet promote stepwise maturation of natural killer cells. Nat Commun (2021) 12(1):5446. doi: 10.1038/s41467-021-25758-2 34521844 PMC8440589

[28] Li N van Unen V Hollt T Thompson A van Bergen J Pezzotti N. Mass cytometry reveals innate lymphoid cell differentiation pathways in the human fetal intestine. J Exp Med (2018) 215(5):1383–96. doi: 10.1084/jem.20171934 PMC5940268 29511064

[29] Hughes T Briercheck EL Freud AG Trotta R McClory S Scoville SD. The transcription factor ahr prevents the differentiation of a stage 3 innate lymphoid cell subset to natural killer cells. Cell Rep (2014) 8(1):150–62. doi: 10.1016/j.celrep.2014.05.042 PMC4133146 24953655

[30] Raykova A Carrega P Lehmann FM Ivanek R Landtwing V Quast I. Interleukins 12 and 15 induce cytotoxicity and early nk-cell differentiation in type 3 innate lymphoid cells. Blood Adv (2017) 1(27):2679–91. doi: 10.1182/bloodadvances.2017008839 PMC5745129 29296921

[31] Spits H Bernink JH Lanier L. Nk cells and type 1 innate lymphoid cells: Partners in host defense. Nat Immunol (2016) 17(7):758–64. doi: 10.1038/ni.3482 27328005

[32] Mjosberg J Bernink J Peters C Spits H. Transcriptional control of innate lymphoid cells. Eur J Immunol (2012) 42(8):1916–23. doi: 10.1002/eji.201242639 22865043

[33] Gordon SM Chaix J Rupp LJ Wu J Madera S Sun JC. The transcription factors T-bet and eomes control key checkpoints of natural killer cell maturation. Immunity (2012) 36(1):55–67. doi: 10.1016/j.immuni.2011.11.016 22261438 PMC3381976

[34] Lazarevic V Glimcher LH Lord GM. T-Bet: A bridge between innate and adaptive immunity. Nat Rev Immunol (2013) 13(11):777–89. doi: 10.1038/nri3536 PMC6290922 24113868

[35] Freud AG Keller KA Scoville SD Mundy-Bosse BL Cheng S Youssef Y. Nkp80 defines a critical step during human natural killer cell development. Cell Rep (2016) 16(2):379–91. doi: 10.1016/j.celrep.2016.05.095 PMC4970225 27373165

[36] Krabbendam L Heesters BA Kradolfer CMA Spits H Bernink JH. Identification of human cytotoxic Ilc3s. Eur J Immunol (2021) 51(4):811–23. doi: 10.1002/eji.202048696 PMC8248192 33300130

