# Supplementary material for: T-BET drives the conversion of human type 3 innate lymphoid cells into functional NK cells
Source: Front Immunol. 2022 Oct 18;13:975778. doi: 10.3389/fimmu.2022.975778 (PMC9623292; doi:10.3389/fimmu.2022.975778)
Supplement: Supplementary file 1 [file DataSheet_1.pdf]

Supplementary Figures

1 Supplementary Figure 1

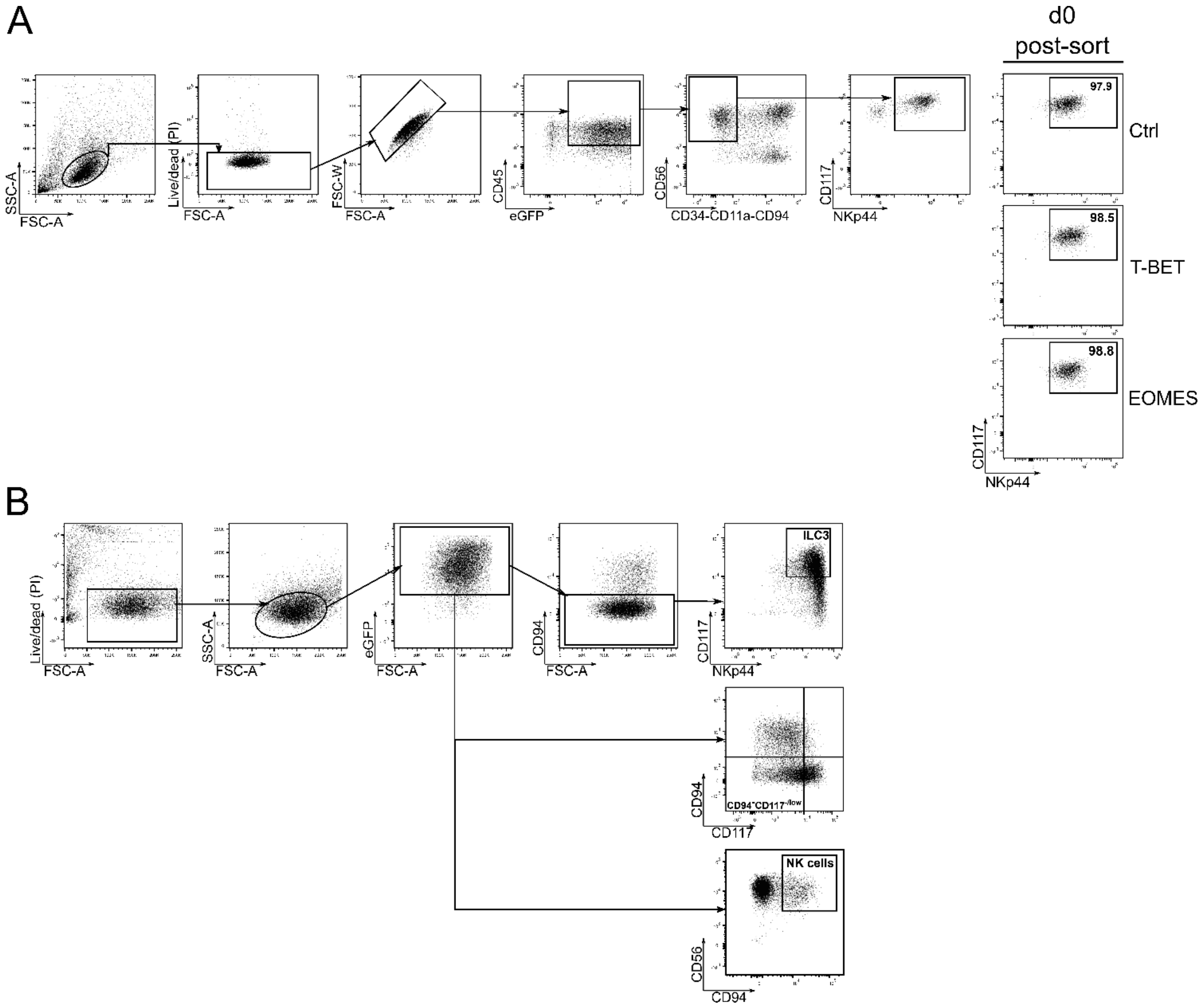

Supplemental Figure 1. Mature ILC3 sort layout and gating strategy for flow cytometry.
(A) Representative dot plots showing the sort layout for isolating mature eGFP$^+$CD45$^+$CD34$^-$CD94$^-$ CD11a$^-$CD56$^+$CD117$^+$NKp44$^+$ ILC3s on day 18 of culture to start the ILC3-NK cell transdifferentiation culture (= day 0). Post-sort ILC3s of the different conditions are demonstrated in the plots on the right. The numbers indicate the percentage of purity. **(B)** Depicted is the representative gating strategy used in flow cytometry to gate the indicated populations in order to evaluate absolute cell numbers and the NK cell phenotype. PI = propidium iodide.

2 Supplmentary Figure 2

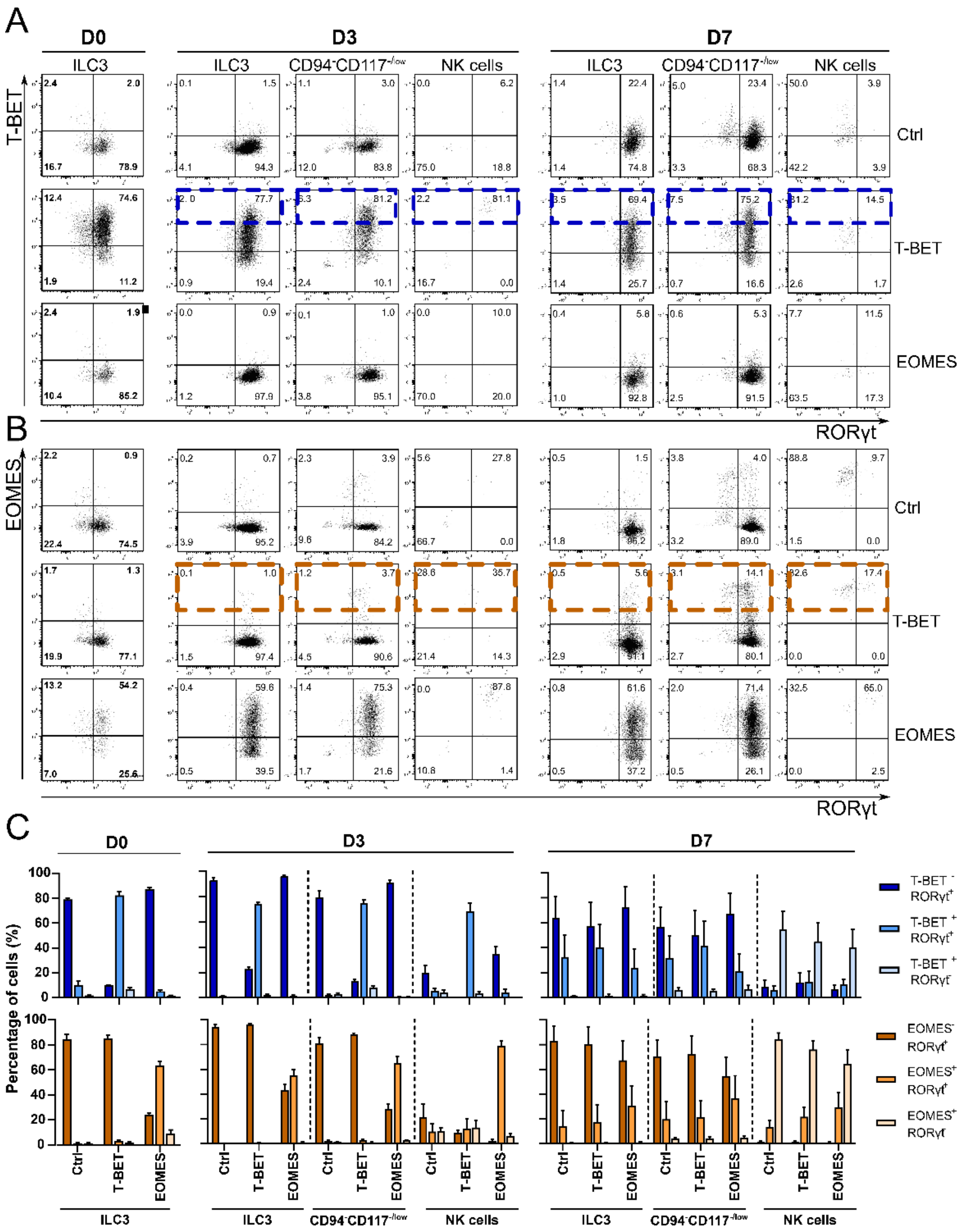

Supplemental figure 2. T-BET, EOMES and RORγt expression during ILC3-NK cell transdifferentiation culture in the absence of 4-OHT. (A-B) Representative dot plots of the indicated populations showing bimodal transcription factor expression on the indicated time points during cultures in the absence of 4-OHT only. **(A)** T-BET vs. RORγt staining. High T-BET-expressing cells in the T-BET-overexpressing condition are highlighted with blue rectangles. **(B)** EOMES vs. RORγt staining. Orange rectangles indicate high EOMES-expressing cells in the T-BET overexpressing condition. The numbers in the plots indicate the percentages. **(C)** Frequencies of bimodal transcription factor expression for the different time points and different populations as indicated (mean ± SEM; n = 4).

3 Supplementary Figure 3

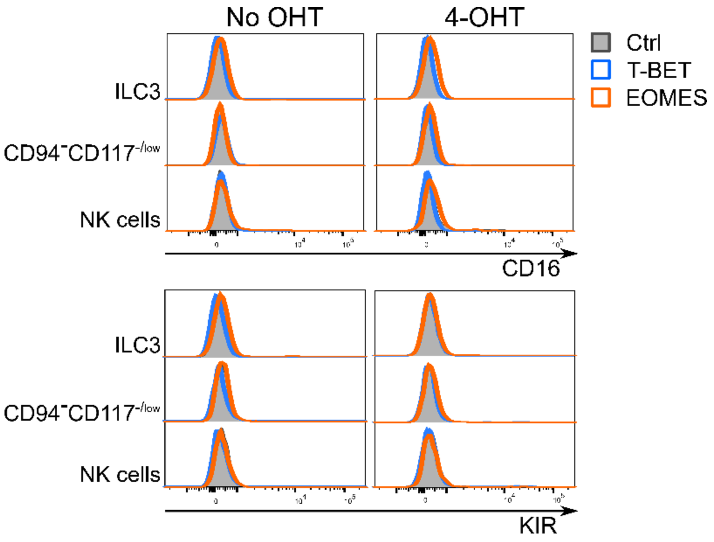

Supplemental Figure 3. NK cells derived from ILC3 lack expression of CD16 and KIR
Pre-gated eGFP$^+$ culture cells from T-BET, EOMES and control conditions in the presence or absence of 4-OHT were analyzed on day 14 by flow cytometry. Shown are the representative histograms of CD16 and KIR expression for the indicated populations.

4 Supplementary Figure 4

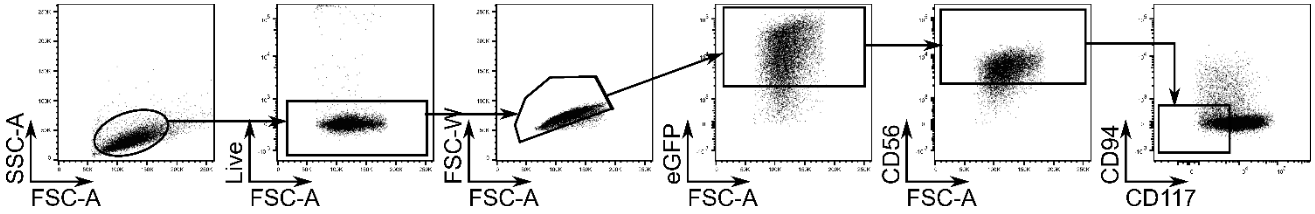

Supplementary Figure 4. $CD94^{-}CD117^{low}CD56^{+}$ sort layout.
Representative dot plots showing the sort layout for resorting $CD94^{-}CD117^{low}CD56^{+}$ cells on day 7 of the ILC3-NK cell transdifferentiation culture to replate these cells and evaluate their NK cell generation potential after an additional 7-day culture.
