# Supplementary material for: T-BET drives the conversion of human type 3 innate lymphoid cells into functional NK cells
Source: Front Immunol. 2022 Oct 18;13:975778. doi: 10.3389/fimmu.2022.975778 (PMC9623292; doi:10.3389/fimmu.2022.975778)
Supplement: Supplementary Table 1 — Antibodies used for flow cytometry. [file Table_1.docx]

Supplementary Tables

# Supplementary Table 1. Antibodies for flow cytometry

| ***Antibody*** | ***Alternative name*** | ***Format*** | ***Clone*** | ***Supplier*** |
| --- | --- | --- | --- | --- |
| CD107a | LAMP-1 | PE | H4A3 | BD Bioscience |
| CD117 | CD117 | PE-Cy7 | 104D2 | Thermo Fisher Scientific |
| CD11a | LFA-1 | Alexa Fluor 700 | HI111 | Biolegend |
| CD11a | LFA-1 | APC | HI111 | Biolegend |
| CD14 | CD14 | APC | 63D3 | Biolegend |
| CD158a/h | KIR2DL1/DS1 | PE | REA1010 | Miltenyi Biotec |
| CD158b1/b2 | KIR2DL2/DL3/DS2 | PE | REA1006 | Miltenyi Biotec |
| CD158e1/e2 | KIR3DL1/DS1 | PE | REA168 | Miltenyi Biotec |
| CD158i | KIR2DS4 | PE | REA860 | Miltenyi Biotec |
| CD16 | CD16 | PE | B73.1 | Biolegend |
| CD19 | CD19 | APC | SJ25C1 | Thermo Fisher Scientific |
| CD3 | CD3 | APC | SK7 | Biolegend |
| CD314 | NKG2D | APC | 1D111 | Biolegend |
| CD335 | NKp46 | PE | 9E2 | Biolegend |
| CD336 | NKp44 | Pacific Blue | 44.189 | Thermo Fisher Scientific |
| CD336 | NKp44 | APC | P44-8 | Biolegend |
| CD337 | NKp30 | PE | P30-15 | Biolegend |
| CD34 | CD34 | APC | 581 | Biolegend |
| CD34 | CD34 | PE | 581 | Biolegend |
| CD45 | CD45 | APC-Fire™750 | 2D1 | Biolegend |
| CD56 | NCAM-1 | Pacific Blue | 5.1H11 | Biolegend |
| CD56 | NCAM-1 | APC-Fire™750 | 5.1H11 | Biolegend |
| CD94 | KLRD1 | PerCP-Cy5.5 | DX22 | Biolegend |
| EOMES | EOMES | eFluor 660 | WD1928 | Thermo Fisher Scientific |
| Granzyme B | Granzyme B | PE | GB11 | Thermo Fisher Scientific |
| IFN-γ | Interferon-γ | eFluor 660 | 4S.B3 | Thermo Fisher Scientific |
| IL-22 | Interleukin-22 | PE | 22URTI | Thermo Fisher Scientific |
| Perforin | Perforin | PE | delta G9 | Thermo Fisher Scientific |
| RORγt | RORγt | PE | AFKJS-9 | Thermo Fisher Scientific |
| T-BET | T-BET | PE | 4B10 | Thermo Fisher Scientific |
